# The Key Role of Strategically and People-Oriented HRM in Hospitals in Slovakia in the Context of Their Organizational Performance

**DOI:** 10.3390/healthcare9030255

**Published:** 2021-03-01

**Authors:** Nadežda Jankelová

**Affiliations:** Department of Management, Faculty of Business Management, University of Economics in Bratislava, 852 35 Petržalka, Slovakia; nadezda.jankelova@euba.sk; Tel.: +421-911-232-927

**Keywords:** healthcare, management, human resources management, mixed human resources management roles, transformational leadership, information sharing, organizational performance

## Abstract

The main objective and purpose of our paper is to verify the positive congruence between the synergistic effect of the mixed roles of human resources management departments in healthcare facilities and their organizational performance. Such congruence is mediated by means of a transformational leadership style and information sharing. The research was carried out on a sample of 44 hospitals in the Slovak Republic, which are included in the ranking according to a comprehensive indicator of their performance (medical and non-medical). Data were obtained using a questionnaire for 44 top managers from these hospitals. Mediation was used as a tool to examine the relevant variables relationship mechanism. All data was analyzed using the SPSS 24.0 software package with the help of selected analytical tools. A series of regression analysis were used to identify the proposed hypotheses. ANOVA was used to analyze the multiple dependence. We worked at a significance level of 5%. The main conclusion of our study is the significant impact of the implementation of the new—mixed role of human resources management departments on organizational performance. Another finding is that the direct effect between the two variables examined is more significant than the mediated effect. This means that if management unambiguously declares and implements the policy of mixed roles of human resources management departments, less influence from the mediator—transformational leadership is sufficient to transmit the effect of this variable onto organizational performance. Completed specialization studies in the field of management play a significant role in the studied relationships.

## 1. Introduction

Recent conclusions from both scientific and professional literature resources draw our attention to the need for a change of the actual human resources (HR) management in terms of shifting the traditional operational role towards a more strategic one. Subsequently, the procedural focus should tend to more people focus approach. Unless such transformation occurs, the HR shall not have the ability to demonstrate and confirm its strategic value within an organization and thus contribute to its overall performance [1,2,3]. Nevertheless, some empirical studies in the healthcare sector suggest that HR professionals generally continue to focus on a more traditional administrative function [4,5,6], which means performing the classic personnel administration in the organization. Our main research concern elaborates on what the shift in human resources management (HRM) in the environment of Slovak hospitals is and whether this shift is related to their performance in terms of their provided service quality, but also patient satisfaction, as well as the economy and transparency of hospitals. We are interested not only in the connection between these two variables, but also in the mechanism through which their mutual effect works. 

### 1.1. Importance of the Study

The topic of our study is important for several reasons. The first reason represents the challenges, which health systems of developed countries are facing today. These challenges are related to demographic change, technologically advanced, comprehensive and costly treatment, growing societal expectations of the healthcare system, coping with patients with chronic diseases and many others that are constantly increasing the need for effectively managed and quality healthcare [7]. 

The second reason represents growing demand of health facilities managers for quality knowledge in management and the related need to saturate this knowledge through the implications of relevant scientific studies. Kuhlman and von Knorring [8] even directly call for the “hybridization” of the relationship between medicine and management, thus to connect two different areas. There is a constant debate in scientific and professional circles about the lack of managerial knowledge and especially the skills of health facilities managers as an addition to their primary highly specialized medical education [9,10,11,12,13]. 

The third reason represents a shift from the use of traditional, paternalistic approach to healthcare delivery towards a patient-centered care approach [14,15], which also requires additional demands for management skills and knowledge. Paternalistic approach is characterized by the complete trust of patients to doctors and their passive role in the treatment process. Subsequently, there is a need to shift HRM departments to strategic partner of the management in order to fulfil such objective. On the other hand, patient-centered medical care may be associated with greater clinician burnout [16], therefore, we consider the need for a shift from management orientation in providing quality, safe and effective health care not only to a patient but especially to an employee. 

A new employee-focused HRM [17] based on job demands-resources model and research about quality of work life [18] has been emphasized for implementation in recent years mainly by modern human resources management, because only a satisfied employee can create added value and contribute to patient’s satisfaction. The job demands-resources model is focused on the analysis of predictors and conditions of psychological wellbeing and stress in the work environment [19,20,21,22,23]. New findings within the job demands—resources model highlight the proactive role of management on the one hand by continuously monitoring and optimizing job characteristics, communicating vision and providing direction and support. On the other hand, it praised the proactive role of employees through adaptive or maladaptive self-regulation strategies [24,25,26,27]. 

The adaptation of employee orientation in individual HR systems of hospitals also results from shifts in national health systems performance (from the so-called Triple Aim to the Quadruple Aim), which, in addition to three main objectives (enhancing patient experience, improving public health and reducing costs), encompasses an additional objective: improving the work life of health care providers, including clinicians and staff [28]. 

Employees’ perceptions about the extent, to which the organization cares about them, are based on relevant HRM policies, holistic organization procedures and the competence of top management to implement these procedures through an appropriate leadership style and transparency of information sharing. In healthcare, this approach is absolutely necessary, considering that the work of healthcare professionals is more about non-financial motivators and missions than about monetary conditions and benefits. Bodenheimer and Sinsky [28] call on health leaders to focus on employee orientation and improve their work life as “the compass point of better care, better health, and lower costs”. 

### 1.2. Literature Review and Development of Hypothesis

The currently available literature in the field of HR health care management contains a low degree of implications from relevant studies on the need to shift the traditional personnel role HRM departments to the level of other important roles it should play in a modern organization. Ulrich [29], Ulrich et al. [30] suggests, that performance can be improved if all their defined HR roles (strategic partner, administrator, employee rights activist and agent of change) are performed simultaneously. The role of the strategic partner focuses on organizational strategies and human resource practices alongside with organization strategy. The human resources administrative expert role is the traditional one. The role of an employee champion includes their involvement in current issues, concerns and staff requirements and the role of change agent refers to the basic change of the culture. All the roles defined by Ulrich are essential for the success of the whole HRM function. Emphasis must be put on all the areas and it is necessary to draw from its synergies. There is no chance to select one and to excel in this one specific area. Many HRM managers forget to balance the approach and they decide to be excellent in one of the needed components and they forget about the danger not meeting the basic requests and expectations in the rest. 

In a later model, Ulrich et al. [30] combined HR competencies and HR roles with organizational performance and substantiated their findings with specific examples from business environment practice. Organizational performance is perceived in this study as a common name for different types of outputs that are positively associated with new HRM roles such as increasing the quality of services provided, increasing innovation, innovative behavior of employees, satisfaction of employees, patients and other stakeholders and much more. Therefore, the central concept of this study is based on the HRM mixed role model [30], according to which the synergistic action of the four roles of HR managers is quintessential to individual and organizational performance. Many other studies have found empirical relationships between the use of modern HRM systems and organizational performance [31,32,33]. Positive effects have also been found between the synergistic effects of multiple complementary practices in HRM and management processes and organizational performance [4,6,34,35,36]. At the same time, many authors [1,2,3,37] state that unless the HRM role is transformed, the HR function will not be able to demonstrate its strategic value within the organization and contribute to overall performance organization. [1,2,3,3,38].

**Hypothesis** **1** **(H1).** *Based on the literature review, we assume that “The HRM mixed role model is positively associated with the overall organizational performance of hospitals”.*

Leaders play a critical role in creating organizational conditions in which employees feel satisfied and in which the HRM mixed role model can be implemented [39,40]. In the field of health care management, transformational leadership has recently emerged as an important factor. Transformational leadership is characterized by such leaders who motivate, inspire the environment by sharing a vision, mission and pay special attention to each individual. Only such leaders can convey the positive effects of the synergistically acting four roles of HRM departments on organizational performance. The findings of the studies point to its connection with the measured output variables. Xie et al. [41] identified a positive relationship between TFL and staff’s job satisfaction and loyalty to the organization. Brown et al. [42], and Lin et al. [43] found that TFL significantly influences intentions to stay, Boamah et al. [44] demonstrated a positive relationship between TFL and patient safety outcomes and Asif et al. [45] found that TFL affects the overall quality of health care. The relationship between effective HR practices and TFL has also been documented in the literature. The authors define these practices through High-Performance Work Practices, which are employee-oriented and highlight the essential role of TFL in their implementation [5,46,47].

**Hypothesis** **2** **(H2).** *We assume that “The HRM mixed role model is positively associated with transformational leadership” and also that “Transformational leadership is positively associated with the overall organizational performance of hospitals”.*

Information sharing is a tool for management that ensures individual and team performance by acquainting employees with the vision, mission and goals of the organization, through clear, timely, regular information about current problems and facts, new intentions and opportunities [48,49]. Only informed employees can contribute to the implementation of changes related to the introduction of new practices in HRM. 

Being a strategic partner of management and at the same time a fighter for employee rights, as well as an agent of change is not possible without knowing the meaning, purpose and importance of this HRM mixed role model within the organization. Bini [50] points to the important role of top management in the ability to implement new roles of HR managers, while top management not only facilitates these changes, but also communicates through vision, strategy and goals. Ward et al. [51] based on the research results, created implications for health care managers to define values and beliefs for employees in accordance with the goals of the organization, which is related to the subsequent implementation of personnel roles in terms of employee involvement in change processes and their strategic management. Information sharing (IS) is presented in many studies as an important mediator in achieving organizational performance by familiarizing employees with the vision, mission and strategic goals of the organization. This should be executed through clear, timely, regular information about current issues and facts [48,52,53,54,55] perceiving managers as internal facilitators and at the same time recognizing the need to develop a structure in healthcare system for an easy and applicable access to information. Moreover, it appears necessary to train managers to accept the role of insider or outsider facilitators of the organization in the healthcare system. Vainieri et al. [56] consider IS to be one of the managerial competencies associated with a higher level of organizational performance. Aragon-Correa et al. [57] even point to a direct relationship between practices that promote information sharing and organizational innovation. Gibson et al. [52] point to the important contribution of information sharing to organizational performance and, at the same time, they have confirmed in their research that information sharing has a unique place among different management practices.

**Hypothesis** **3** **(H3).** *We assume that “The HRM mixed role model is positively associated with information sharing” and also that “Information sharing is positively associated with the overall organizational performance of hospitals”.*

### 1.3. Theoretical Model of the Study

**Figure 1:**
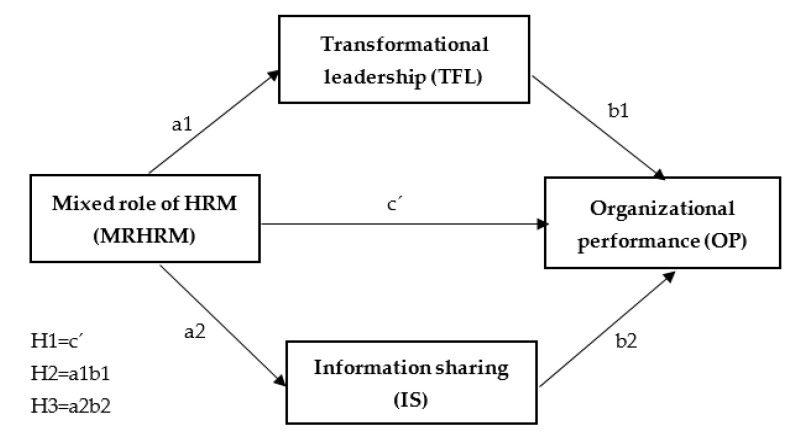
The mediation model and the 3 tested hypotheses.

Based on a literature review and justification of the importance of the topic, we see a large research gap in the study of selected contexts and define the main hypothesis, representing the main purpose of our paper on the positive relationship between the HRM mixed role model in healthcare facilities and their overall organizational performance, which is mediated by transformational leadership and information sharing. Figure 1 shows the model used to test the relationships between variables. 

## 2. Materials and Methods 

### 2.1. Sample and Data Collection

The study is a cross-sectional study. It was performed in a specific time on a sample of different managers of different hospitals. Our sample consisted of 44 top managers of medical facilities in Slovakia. Hospitals, 11 teaching and 33 general hospitals (27 state and 17 private) were selected. The main reason is that only these facilities have published performance indicators in six areas (three medical and three non-medical). These performance indicators are collected and presented by the non-profit non-governmental organization INEKO as an official document of evaluation of hospitals in Slovakia between the years 2015 to 2019, using complex methods of data collection and processing. There is a total of 17 teaching hospitals in Slovakia and 53 general hospitals (excluding specialized hospitals and institutes). In our research were involved 11 of teaching hospitals and 33 of general hospitals. The remaining 6 teaching and 20 general hospitals could not be included in the research due to the fact that they did not pass the qualification criteria of data complexity at the national level and are not presented in the above Slovak ranking of hospitals. A specific calculation of the summary indicator of hospital performance is given in the Measurements section. We also contacted the top management of these facilities in person or by telephone and explained our intention and research model, offering to provide results and comparisons in the field with other facilities in the ranking. After an agreement with the representatives of these facilities, we sent them a questionnaire. The study adopted a cross-sectional web-based survey design distributed via e-mail. The questionnaire was sent at the end of September 2020. By the end of October, all 44 responses were returned. The return was 100% due to the fact that we contacted only pre-agreed contacts, as the output information on organizational performance was available only from the selected hospitals. The questionnaire contained identification data at the beginning and the core of the questionnaire consisted of scaled questions. Respondents are hospital top managers, who are the key persons for shaping the position and operation of the entire structure of HRM in the hospital. 21 correspondents are non-medical university graduates, 20 respondents completed managerial specialization studies and in terms of gender variety the sample consisted of 9 women and 35 men. 

### 2.2. Measures

Mediation was used to test the relationships between MRHRM, OP, TFL and IS, which we consider to be a suitable tool for a deeper examination of the relationships between variables and the mechanism on the basis of which these relationships work. 

MRHRM is an independent variable that is operationalized as a score, created based on managers’ answers to questions related to the fulfilment of individual roles of HRM departments in their organizations. The methodological starting point is the model of mixed roles of personnel departments, presented by Ulrich [25], which is a tool in determining the orientation of HRM and can also serve for the needs of self-assessment in this area. The instrument is not validated and is developed ad hoc. Ulrich’s model of HRM distinguishes between strategy and operations and people and process in HRM roles. The model contains 40 statements, divided into ten areas. Each area therefore contains four statements, each statement falling into one of the four roles of HRM departments, namely a strategic partner (example of statements—e.g., HRM department helps to fulfil the tasks of the whole hospital, HRM department participates in the process of defining hospital strategy), personnel administration (example of statements—e.g., HRM department ensures effective organization of processes in personnel work, HRM department spends most of its time on operational matters), employee rights activist (example of statement—e.g., HRM department helps to take care of employees’ needs, HRM department participates in improving commitment and employee engagement) and the change agent (example of a statement—for example, the HRM department helps to adapt to change, the HRM department is involved in creating a change in organizational culture). In total, the independent variable MRHRM contains 40 items (in Appendix A), which are scaled using a five-point rating scale (1—the lowest rating, 5—the highest rating), while the number of points is added for each role. This means that the maximum number for a single role is 50 points and for all roles at the same time 200 points. After reliability analysis, the Cronbach’s alfa of the MRHRM was 0.94 (40 items). 

By confirmatory factor analysis, completed by a statistical test of the hypothesis of the suitability of the selected factor structure, we confirmed the defined factor structure within the MRHRM variable, where partial items are saturated with four different factors. Nevertheless, they can be used as a whole, because the assignment we assumed is one of the possible, so it is not unique (the CFI coefficient that compares the assumed model with the worst possible baseline model was 0.76; the Chi2 *p*-value was 0.000). Other criteria of confirmatory factor analysis were satisfactory (assignment—one item—one factor; signs for factor saturation (positive/negative)—all saturated positive, coefficient SRMR = 0.052; RMSEA = 0.077). 

The second variable represents the indicator of the OP of the medical facility. It is a composite indicator, composed of six sub-indicators—the quality of health care provided, the experience of hospitals, the complexity of diagnoses, patient satisfaction, management and transparency. These indicators are monitored and published by the non-governmental non-profit organization INEKO (Institute for Economic and Social Reforms of the Slovak Republic), which evaluates health care facilities based on the established methodology since 2015, accepting their availability and relevance, wide scope and stability. Several years so as to minimize the impact of random one-off fluctuations, a 4-year period was observed for most indicators). INEKO collects data from health insurance companies (General Health Insurance Company Slovakia, Health Insurance Company “Dôvera”, Union Insurance Company), health facilities, the Ministry of Health of the Slovak Republic, the Ministry of Finance of the Slovak Republic, self-governing regions, the Health Care Supervision Office, the National Health Information Centre, the Emergency Medical Service Operations Centre and Transparency International Slovakia. The evaluation is carried out for state university and university hospitals—11 facilities (note: children’s university hospitals were not assessed) and general hospitals—53 facilities (of which 33 hospitals passed the qualification criteria). The first indicator is the quality of health care provider (sub-indicators: reoperation, total rehospitalization up to 30 days, mortality after operations, mortality from acute cerebrovascular accident, mortality after femoral fracture (65+ years), mortality in the intensive care unit, mortality from in the inpatient department after translation from the intensive care unit, waiting time of the patient for emergency admission brought by the ambulance, and finally fines from HCSA (Health Care Surveillance Authority) (weight 40%). The indicators result from the statutory quality indicators in the field of health care outcomes. Indicators and their definition are determined by the Ministry of Health of the Slovak Republic. Health insurance companies are required to monitor these indicators. The data is drawn from the healthcare provided to them show individual providers. The second indicator—experience (sub-indicators: Index of the number of so-called EBHR procedures (procedures used in stratification; weight 10%). It is a summary indicator consisting of the evaluation of various groups of procedures stratification of hospitals. The third indicator—the complexity of diagnoses (sub-indicators: Case Mix Index (CMI) of the hospital, expressing the average economic and medical intensity of patients hospitalized in the hospital for a certain period of time, in our case per year; weight 10%). The fourth indicator is patient satisfaction (sub-indicators: overall patient satisfaction and patient complaints; weight 18%). It is a summary indicator—the average of 12 statutory quality indicators in the field of perception of healthcare provision by hospitalized patients. The indicator is formed as a synthetic index of the subjective evaluation of the provider from the point of view of patients covering the evaluation of their satisfaction with care, behavior and information provided by health care staff, evaluation of accommodation quality, cleaning of wards and diet and evaluation of satisfaction with provided care and subjective perception of treatment success. Complaints are measured as the total number of complaints per hospital in relation to 1000 hospitalized patients, which were addressed to the Health Care Supervision Office and where the Office terminated the supervision of the provider concerned. Fifth indicator—management (sub-indicators: ability to generate own funds and overdue debt and its year-on-year change; weight 12%) and the sixth indicator—transparency (sub-indicators: transparency index representing a summary evaluation of individual facilities based on the level of quality of information for patients and others public and economic information; weight 10%). The final evaluation of the hospital is calculated as a weighted average of the points achieved for the above indicators. In total, the hospital facility could get a maximum of 100 points, a minimum of 0 points, with the more points, the better the rating and ranking. 

The third and fourth variables are intermediate variables—mediators. The first mediator is the TFL. This variable is operationalized as an expression of managers in relation to the four dimensions of TFL—intellectual stimulation, inspiring motivation, idealized influence, individual approach, which were measured using a 20-item scale developed by Bass and Avolio [58] mentioned in Appendix A. The Multifactor Leadership Questionnaire is a proven and frequently used tool for evaluating the transformational style of leadership and is considered the best validated measure of this style [59]. As the MLQ questionnaire is not translated into Slovak, we worked on its translation for the purposes of our research. The questionnaire was translated from the English original into Slovak by two independent persons. Both translations have been summarized and re-evaluated. The resulting version of both translations was back-translated into English by a third party for comparison. The resulting translation was used in our research. Responses to individual items within the TFL characteristics were scaled on a 5-point scale (1 = “very seldom” to 5 = “very frequently”). After reliability analysis, the Cronbach’s a of the TL was 0.85 (20 items). 

The second mediator is IS. This variable is operationalized as a score created based on managers’ statements on items adopted from the study of Ketokivi and Castañer [60], who measured the sharing of general information and communication about organizational priorities with employees. In total, the intermediate variable IS contains 5 items (e.g., management regularly informs employees about important changes, management regularly informs employees about overall policies and objectives), included in Appendix A, which are scaled using 5-point Likert-type scales (5—I completely agree, 1—strongly disagree). After reliability analysis, the Cronbach’s alpha of the IS was 0.95 (5 items). 

The internal consistency of the all examined variables (MRHRM, OP, TFL and IS) used is very good. It is greater than 0.7 for all measurements. 

### 2.3. Data Analysis 

All data was analyzed using the SPSS 24.0 software package. Cronbach’s Alpha coefficient was used to assess the internal consistency of the scale’s reliability. In order to eliminate the detrimental effects of method biases, we have used one of the statistical remedies, namely factor analysis [61]. To verify the factor structure of mediating variables, the CFA was facilitated. We verified factor structure using scattering factor fixation method in order to determine free-covariation factor coefficients. The factor-based average score method was used as a means to calculate factor score. Based on Hofmann’s [62] suggestion, we conducted a hierarchical regression analysis to test the mediating effect. Additionally, we followed Baron and Kenny’s [63] procedure to test the stated mediating effect. The Sobel Test was used to test the mediator effect. A series of regression analyses was used to identify the proposed hypotheses. The ANOVA variance analysis was used to analyze multiple dependencies. We have worked with a 5% significance level 

## 3. Results

### 3.1. Descriptive File Analysis and Context Identification

Relationships between individual variables were determined using a correlation matrix, which also includes control variables (Table 1). 

Control variables were ownership (private, state), gender, education, specialization in management, which were selected as control variables given their theoretical relevance. In private hospitals, a higher implementation of modern management tools is observed in order to increase their performance [64,65]. In our sample, a significant correlation was not confirmed. Gender was correlated negatively especially with strategic HRM and behavior of HR managers, then the change agents indicating, that females reported higher levels of these aspects of HRM than males did. According to our correlations, the opposite effect has been shown. However, it is not significant. Managerial specialization in health care managers positively affects the performance of their managed organizations [11], the combination of medical professional education and further management education has the greatest impact on the positive relationship between modern human resource management and organizational performance [8,66]. A significant and relatively high positive correlation coefficient was also demonstrated in our study in connection with all examined variables. Organizational size was correlated negatively [4]. However, we did not address this variable because all the surveyed hospitals were large in terms of size. The table also provides brief descriptive statistics. 

It is clear from the correlation matrix that there are significant positive correlations between all the variables examined, indicating the use of a mediation model. However, we also see a significant relationship between completed specialization studies in management and all variables (OP, MRHRM, TFL and IS), which is a significant finding in terms of the need for this education in the ranks of health care managers. At the same time, descriptive statistics point to individual descriptive values of the file. For the OP variable, the minimum value is 37 and the maximum 71 out of the total possible number of 100 points (average = 52.14, SD = 6.70). For the variable MRHRM, the minimum value is 81 and the maximum is 153 out of the total possible number of 200 points (average = 122.7, SD = 17.8). The highest average value was found in the role of personnel administrator (average = 43 points), followed by the role of a fighter for employee rights (average = 37.8 points), in which personnel provide and manage their contribution. We expected such a higher average, because in general, this role is, according to the creators of the methodology, the most preferred by many organizations and usually well provided. In this position, HRM departments address the issue of employee contribution and its maintenance at a stable high level through the balance of employee demands on the one hand and the possibility of their implementation on the other hand. The other two roles had a lower average rating, namely the strategic partner (average = 21.1 points) and the agent of change (average = 20.9 points). 

Mediation variables that were rated on a Likert scale from 1 to 5 (1—strongly disagree, 5—strongly agree) were the higher average (3.71) found for the mediation variable IS with a lower standard deviation, indicating its greater significance compared to the TFL variable where an average of 3.30 with a higher SD of 0.57 was found. Simple correlations between IS and the variables OP and MRHRM are lower than between TFL and the variables mentioned. In both cases, they are positive and significant. 

### 3.2. Mixed Role of HRM Departments as Predictor of Organizational Performance

By mediation we want to test whether a third variable (TFL and IS) explains the relationship between predictor and outcome in the form of an indirect effect. In mediation, we proceeded from the established main hypothesis, which applies when the indirect effect is significant using the Sobel test. We added control variables, education, completed specialization and ownership to the modelling of the overall effect. ANOVA was used as an intermediate step in the analysis of multiple dependence, where we found that none of the above control variables is significant. 

Subsequently, we proceeded in three steps (A, B, C), in which we verified partial hypotheses by calculating three regressions. The steps examine the following relationships, expressed in Models 1 through 4, shown in Summary Table 2: 

(C) There is a relationship between OP (variable Y) and MRHRM (variable X). 

(A) There is a relationship between the mediation variables TFL (variable M1) and IS (variable M2) and MRHRM (variable X). 

(B) There is a relationship between OP (variable Y) and the mediation variables TFL (variable M1), IS (variable M2), in which MRHRM (variable X) does not participate. 

The value of C represents the total effect. The product A * B is a mediated (indirect) effect of X on Y through M (due to the existence of two mediation variables, the mediated effect is expressed in the form A1 * B1 + A2 * B2 + A1 * B2 * D21, where member D21 is the path from M1 to M2). The difference C‘ = C—indirect effect is the pure (direct) effect of X on Y without the participation of M. The hypothesis holds when the indirect effect is significant. Using the Sobel test (A * B = 0.391, z = 0.751, SE = 0.035, Sig. = 0.000), we found that the overall indirect effect is significant in the positive direction. We present the effects in a standardized form. Standard errors are calculated from the bootstrap method with 5000 repetitions. 

From the results in Table 2 it is clear that the overall effect (C) is significant and the dependence is positive (model 1, coef. = 0.959, Sig. = 0.000), which indicates the existence of a relationship between OP in facilities and MRHRM. Step A is significant, so there is a relationship between the mediation variable TFL and MRHRM (model 2, coef. = 0.643, Sig. = 0.000) and at the same time, due to the realization of serial mediation, there is a relationship between both mediation variables (D21)—model 2, coef. = 0.350, Sig. = 0.000). Furthermore, there is a relationship between MRHRM and the mediation variable IS (model 3, coef. = 0.740, Sig. = 0.000). The direct effect (C ‘), i.e., the effect without the participation of mediating variables, is significant (model 4, coef. = 0.568, Sig. = 0.000). Step B, expressing the relationship between OP (dependent variable Y) and mediation variables (M1 and M2) in the form of TFL and IS, in which the dependent variable X (MRHRM) does not participate, is significant in part only for the variable TFL (model 4, coef. = 0.375, Sig. = 0.000). IS coef. = 0.071, Sig. > 0.005) means an insignificant dependence. The total indirect effect A * B thus arises very low, namely 0.391 with size z = 0.751, at the same time with significance Sig. > 0.005, which means that mediation variables as a whole do not have a significant indirect effect in the relationship between MRHRM and OP facility. 

The obtained results show that the OP of Slovak hospitals is mainly influenced by the independent variable MRHRM in the form of a direct effect, acting in a positive direction. Its action is influenced by the TFL mediator only to an insignificant extent. Due to the insignificant action of the second mediator, which act serially in the mediation, there is a situation that the overall indirect effect is insignificant. When measuring the size of individual effects as a percentage, based on the obtained coefficients, we state that the size of the direct effect is 59% (coef. = 0.568) and the size of the indirect effect is 41% (coef. 0.391). The relationship between MRHRM and OP hospitals is largely mediated by the direct relation of these two variables. TFL with a significant degree of effect is also involved in the relationship to some extent, which, however, IS in serial mediation is attenuated and do not show a statistically significant indirect effect as a whole. 

Due to this fact, we statistically verified the mediation either the existence of only one mediation variable, namely TFL, while the indirect effect in this case, verified by the Sobel test was significant (A * B = 0.853, z = 1.324, Sig. = 0.000), which means incomplete mediation at 57% direct effect and 43% indirect but significant TFL effect. 

**Figure 2:**
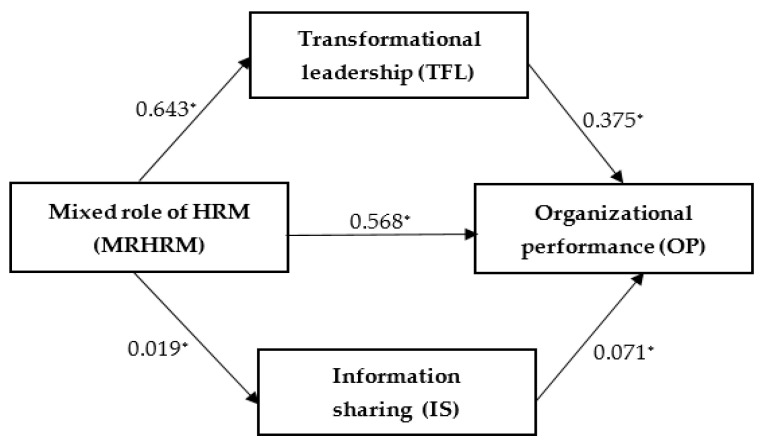
Estimated standardized path coefficients. Note: (*) statistically significant result at a significance level of 5%, i.e., *p* < 0.05.

The empirical model with estimated value is shown on Figure 2. 

## 4. Discussion

### 4.1. Main Findings and Interpretation in Perspective of Previous Studies

It is clear from the above that a more operative and procedural approach in HRM prevails in Slovak hospitals and shifts are necessary in the strategic level of its perception and people orientation. The role of the strategic partner is reactive and does not belong to the dominant aspects of HRM departments in hospitals, there is a lack of understanding of its meaning by the management itself. Its role should be to proactively formulate challenges to senior management and to participate fully in formulating the long-term goals of the organization. The role of the agent of change is also underestimated, while changes are already becoming a permanent part of the provision of health services and occur much more often than in the past. HRM departments can significantly support the success of the implementation of organizational change. It can be both a professional support in the implementation of change, but also an active implementer of changes due to the possibility of a wide impact on employees. 

No previous research in the conditions of Slovak healthcare has been conducted exploring relations and the mechanism of cooperation between HRM departments and the management of organizations in the context of the performance of healthcare facilities. The study addresses this shortcoming in the literature and, to the best of our knowledge, is the first study to examine such a combination of factors, with organizational performance not just being a subjective expression of managers but a comprehensive indicator based on a wide range of data obtained from a wide range of stakeholders. Despite some empirical confirmation of the relationship between the HRM mixed role model and performance, there is no consensus as to the mechanisms that explain this connection. Evans & Davis [67] and Pereira & Gomes [68] suggest that the social context plays an important role in this relationship, as it is necessary to influence employees‘ sensemaking. 

The main finding of our study is the significant impact of the implementation of the new—mixed role of HRM departments on the overall organizational performance of hospitals. HRM department, if it fulfils its main and basic role of personnel administrator, can fulfil the roles of management partner in the field of people management, especially the role of employee of the strategic partner and change agent. This significantly contributes to higher organizational performance in terms of quality of services, patient satisfaction, economy and transparency. Another finding is that the direct effect between the two variables examined is more significant than the mediated effect. This means that if management unambiguously declares and implements the HRM department mixed role policy, less influence from the mediator—transformational leadership, is sufficient to transmit the effect of this variable on the overall organizational performance of hospitals. Even, information sharing as a serial mediator transmits the mediated effect to the level of insignificance. This is an interesting finding of our study, as information sharing is highlighted by many authors as an important support tool within various innovative management tools [51,56]. Even within the new understanding of the job demand-resources model, information sharing, communication of vision, goals and changes is an important factor in the proactive role of management to optimize demands and resources [27]. 

Although a simple correlation revealed significant positive partial connections between information sharing and the overall organizational performance of hospitals and also between information sharing and the HRM mixed role model. Thus, their direct effect on the examined variables is obvious, nevertheless, it did not prove to be significant in mediation, which indicates the strong position of an effectively functioning HRM mixed role model. The indirect effect was significant only in transformational leadership, however, it was lower than the direct effect. Transformational leadership transmits a partially positive effect between the HRM mixed role model and the overall organizational performance of hospitals. Of the four components of the transformational leadership, the most significant influence is the idealized influence, characterized by a high self-confidence of the manager in their competence, devotion to their own opinions and ideals, as well as a certain degree of charisma. All this reinforces the trust of employees and creates a sense of stability. By their actions, managers set subordinates an example to be followed, clearly specify the need for strong commitment in achieving goals and give a higher sense of work, encouraging enthusiasm and commitment to common goals. This result is in line with discussions regarding how trust in management and a strong idealized influence of leaders can result in better organizational results [69,70,71,72,73] and how psychological mechanisms within transformational leadership enable the leader to influence employees’ perceptions [68,74,75]. Another conclusion of our study is also the fact that the connection between the HRM mixed role model and the overall organizational performance of hospitals, mediated by transformational leadership, was more significant among managers with a completed specialization study in management, which contributes to the discussion about the need for managerial education for health managers. Recently, these discussions have been rather frequent and imply the need to transform the roles of health managers in order to change the quality of services provided [76], the ability to manage financial and operational resources and social performance [77], as well as the approach to employees resulting in greater satisfaction [78,79]. 

In the conditions of the Slovak Republic, these discussions come to the fore, as the obligation to complete managerial specialization studies was legally abolished in 2018 and replaced only by sufficient fifteen years of management experience. Foreign studies show a high level of attention paid to the training of managers and health leaders [80]. Programs aimed at training leaders in health care were launched mainly in response to internal study plans that were underdeveloped or lacking in medical studies. Careau [81] in his study reviewed 250 training programs for health care executives and identified the most important topics to be addressed, such as leadership styles, communication skills, emotional intelligence, building a supportive organizational culture, and more. Zakariasen a Henderson [82] highlight the education of managers directly in the health care organization, while seeing the added value of such education in increasing the efficiency of system-wide processes. Californian scientists Hopkins et al. [83] even evaluated the effectiveness of health managers’ training programs through the Kirpatrik model. Training of managers and HR managers is very important for the complex needs of changes within the health care system. 

### 4.2. Implications for Healthcare Management

Our findings may have important implications for practice. First, our study showed that the shift in HRM towards a strategic approach and towards employees in the form of partnership is reflected in organizational performance and has a direct impact on the quality of health services provided, patient satisfaction, hospital management and transparency. 

If HR departments want to make full use of the potential they have and thus contribute to creating value for the organization and the services it provides, they must perform their roles as a whole rather than individually. Some of the roles are basically contradictory and enable to somehow balance the whole system. The need for change and innovation (agent of change) within organizations seems to be balanced by stability and continuity (personnel administration). The role of a strategic partner, where HR professionals stand on the side of the management and defend its interests, is in contrast to the role of the employee advocate, when, on the contrary, they interpret the opinions of employees to the management. The role must be, therefore, understood and performed as the so-called mixed rolls. 

In order to achieve a balance within the individual roles, an increased orientation of HRM departments towards the perspective roles of a strategic partner and agent of change is needed. The imbalance that is common in the hospitals studied is a serious drawback at a time when people management is perceived primarily through its contribution to business results, in contrast to the traditional approach, which perceived this area as a service background and focused primarily on content and processes in this area. Competitive and comprehensive people management acquires a strategic role and generates an added value for all parties involved—the organization, its employees, and last but not least, the patients. The growing pace of change in all relevant environments of interaction with the organization raises the need to overcome the established views and patterns of behavior that were created and functional in the past, but seem, however, insufficient today. 

Transformational leadership transmits a part of the effect of the HRM mixed role model on the overall organizational performance of hospitals. It is necessary to focus on the use of those aspects of the transformational style of management, which mean confidence, stability and security for employees in the scope of the ever-increasing demands of the current work environment and its turbulence. 

Healthcare HR managers play an irreplaceable role in ensuring high organizational performance. There is a need to establish a pressure for legislative support to implement the solid management education within the entire health care system. The topic of HRM should be more explicitly and methodically implemented in the Slovak healthcare curriculum in specialized studies for healthcare managers. 

### 4.3. Limitation of the Study

The presented study has several limitations. The first of them is the sample of respondents (44) and the geographical limitation of the study to the territory of the Slovak Republic. The sample contained only faculty and general hospitals, so it is not possible to generalize the findings to all subjects in health care. 

Due to the real existing data on the performance of Slovak hospitals, it was not possible to use a larger sample of hospitals. The second limitation is the one resulting from the fact that in the modelled relationships we deal only with the connections between the variables. To claim causality, we lacked two conditions, namely accrual and exclusion of another possibility (we had this condition partially fulfilled by controlled effects, but not completely, as our data were not experimental, but questionnaires and formed a “so-called convenience sample”). Therefore, we did not address these issues. In the future, our research can be moved to the level of causality research using dynamic panel regression, which will allow us to take into account the existence of endogeneity and more appropriately describe the ongoing process of adaptation over time as in the case of a statistic panel. The third limitation might be the overestimated and subjective view of top managers on their management processes. 

Consequently, future research can focus on the views of other parties providing HRM in hospitals, or the views of employees. Finally, in addition to the factors concerned in this study, there might be other factors that may affect the examined relationships, for example the training of managers as a mediating factor, but also social responsibility, community engagement to support sustainable changes in the health sector care. 

In the future, other theories can be combined and a comprehensive analysis can be performed from various perspectives. At the same time, when the ranking of hospitals is enriched with other subjects, the research can be carried out subsequently in these hospitals as well. 

## 5. Conclusions

The mixed role of HRM departments means the synergistic action of four areas in the people management with a trend resulting in their shift from supervision to partnership, from processes to people, from administrative to consultative or from operational to strategic approach. Nevertheless, we consider it necessary to state that such a perception and setting of human resources management presupposes perfect mastery of the expert role, which then creates a necessary basis for the expansion and a transformation of the entire human resources management in this direction. The aim of this paper was to verify the hypothesis of a positive relationship between the implementation of the mixed role of HRM departments in healthcare facilities and their overall organizational performance, which is mediated by transformational leadership and information sharing within an organization. The hypothesis was partially confirmed, because sharing of information reduced the indirect effect in the investigated mediation. Transformational leadership conveys this effect. However, the direct effect of the HRM mixed role model is strong and suggests the importance of a correct understanding of HRM within organizations. HR managers might perform strategic as well as operational roles in the hospital, but these tasks need to be more strategy-focused and people-oriented. However, the fundamental principle is a transformational style of management leadership that will support the performance of these roles. Our contribution and our findings expand the field of knowledge in management, in the healthcare and healthcare management. Consequently, we point out that an international cooperation of researchers in this field is necessary in order to further develop the knowledge base of the international scientific community. 

## Figures and Tables

**Table 1:** Descriptive statistics of variables and correlation matrix.

Variable	N	Mean	SD	OP	MRHRM	TFL	IS	Education	Specialization	Gender
OP	44	52.14	6.70	-						
MRHRM	44	122.73	17.85	0.959 **	-					
TFL	44	3.30	0.57	0.946 **	0.902 **	-				
IS	44	3.71	0.47	0.801 **	0.740 **	0.825 **	-			
education	44	0.48	0.51	−0.243	−0.199	−0.227	−0.205	-		
specialization	44	0.32	0.47	0.736 **	0.719 **	0.672 **	0.501 **	−0.360 *	-	
gender	44	0.20	0.41	−0.052	−0.059	−0.046	0.460	0.305 *	0.500	-
Legal form	44	0.61	0.493	0.968	0.937	0.737	0.436	−0.593	−0.059	0.253

Note: OP = organizational performance, MRHRM = mixed role of human resources management departments, TFL = transformational leadership, IS = information sharing, education (medical = 0, other = 1), gender (male = 0, female = 1), specialization = specialization in management (yes = 1, no = 0), ownership (private = 0, public = 1). ** Correlation is significant at the 0.05 level (2-tailed), * Correlation is significant at the 0.01 level (2-tailed).

**Table 2:** Regression results for main effects and mediation analysis.

Variable	Model 0			Model 1			Model 2			Model 3			Model 4		
Dependent	OP			OP			TFL			IS			OP		
	C	SE	S	C	SE	S	C	SE	S	C	SE	S	C	SE	S
Constant	10.674	2.763		7.973	2.042		−0.820	0.264		1.342	0.337		7.686	2.010	
Main effects															
MRHRM	0.334 *	0.024	0.891 *	0.360 *	0.016	0.959 *	0.021 *	0.003	0.643 *	0.019 *	0.003	0.740 *	0.213 *	0.029 *	0.568 *
IS							0.428 *	0.103	0.350 *				1.017	0.841	0.071
TFL													4.393 *	1.069 *	0.375 *
**Controls**															
education	0.041	0.660													
specialization	0.085	0.958													
gender	0.027	0.772													
ownership	0.024	0.615													
R2=	0.916			0.917			0.862			0.536			0.953		

Note: OP = organizational performance, MRHRM = mixed role of human resources management departments, TFL = transformational leadership, IS = information sharing, education (medical = 0, other = 1), gender (male = 0, female = 1), specialization = specialization in management (yes = 1, no = 0), ownership (private = 0, public = 1). R2.adj—adjusted coefficient of determination, C= unstandardized coefficient B, SE—standard error of the estimate, S = Standardized, (*) statistically significant result at a significance level of 5%, i.e., *p* < 0.05.

## Data Availability

The data presented in this study are available on request from the corresponding author, N.J. The data are not publicly available due to restrictions e.g., their containing information that could compromise the privacy of research institutions.

## Appendix A

Mixed roles of HRM departments

- The department of HRM helps the organization:
  fulfil the tasks of the entire organization, improves the efficiency of operations, caters for the personal needs of employees, adjusts to change.
- The department of HRM contributes to:
  the process of defining organizational strategies, the creation and maintenance of processes in the field of HR, the improvement of employee loyalty and engagement, the changes in the organizational culture to help innovation.
- The department of HRM ensures:
  the interconnection between HR strategies and the strategies of the entire organization, the efficient organization of processes in HR operations, the harmonization of HR policy with the personal needs of employees, the promotion of abilities of the organization to execute changes.
- What is your opinion about the effectiveness of an HRM department? It is effective, if it is able to:
  facilitate strategy implementation, guarantee the efficient functioning of HR processes, help employees in satisfying their own needs, support the organization to anticipate future challenges and adapt them.
- Do you consider, or perceive the department of HRM as:
  a strategic partner in office management, an expert in HR administration, a champion for workers, a partner in the implementation of changes?
- The department of HRM in the organization devotes the most time to:
  strategic matters, operational matters, problems with workers, listening to them and finding solutions, the promotion of behavioral changes to improve the quality of the organization.
- The department of HRM is an active participant in:
  organizational planning, creating and maintaining the processes of HR operations, reacting to the problems of workers, renewing or changing the organization.
- The department o HRM aims to:
  connect and harmonize the overall policies of the office, monitor the administrative processes, offer assistance to workers in satisfying family and personal needs, mold the behavior of employees to promote organizational change.
- The department of HRM aims to:
  connect HR strategy with the implementation strategy of the organization, effectively process documents and agreements, respect the personal needs of employees.
- The credibility of the HRM department is built through:
  promoting the fulfilment of the strategic objectives of the organization, increasing productivity, supporting workers to meet their own needs, the efforts to implement changes.

Transformational leadership

Idealized influence

(a) I encourage employees to feel proud to be able to work with me.
(b) I sacrifice my personal interests for the good of the group.
(c) I act in such a way that others respect me.
(d) I show strength and self-confidence.
(e) I am talking about my most important values and beliefs.
(f) I clearly specify the need for strong faith in achieving goals.
(g) I consider the moral and ethical implications of my decision.
(h) I emphasize the importance of a common perception of the mission.

Intellectual stimulation

(a) When making critical comments, I examine whether they are justified.
(b) I am looking for other ways to solve problems.
(c) I give others an insight into the problems from many angles.
(d) I suggest new ways how to task to complete.

Inspiring motivation

(a) I am optimistic about the future.
(b) I talk enthusiastically about what is to be achieved.
(c) I am convincingly formulating a vision of the future.
(d) I express my faith in the successful achievement of goals.

Individual approach

(a) I spend time learning and coaching others.
(b) I treat people rather than individuals rather than group members.
(c) I am aware that each individual has individual needs, abilities and ambitions.
(d) I help others develop their strengths.

Information sharing

(a) In our hospital, the hospital’s management regularly informs employees about important changes.
(b) In our hospital, the hospital’s management regularly informs employees about overall policies and goals.
(c) In our hospital, the hospital’s management regularly informs employees about the method of evaluating the hospital’s performance and about the achieved results.
(d) In our hospital, the hospital’s management regularly informs employees about the plans of its departments.
(e) In our hospital, the hospital’s management regularly informs employees about the requirements concerning the performance of their work.
